# ‘Family court…sucks out your soul’: Australian general practitioners’ experiences supporting domestic violence survivors through family court

**DOI:** 10.1186/s12875-023-02044-2

**Published:** 2023-04-11

**Authors:** Jacqueline Kuruppu, Kitty Novy, Lily Fetter, Sanda Oo, Kelsey Hegarty

**Affiliations:** 1 Department of General Practice, The University of Melbourne, Melbourne, Australia; 2 Safer Families Centre of Research Excellence, Victoria, Australia; 3 The Royal Women’s Hospital, Victoria, Australia

**Keywords:** Domestic violence, Intimate partner violence, Primary care, Family court

## Abstract

**Background:**

Domestic violence is a significant public health issue with survivors experiencing short- and long-term physical, sexual and psychological health issues. Given this, survivors of domestic violence use healthcare services at an increased rate compared to the general population. Therefore, general practitioners (GPs) are well placed to support survivors of domestic violence. However, many practitioners do not feel ready to address this complex issue of domestic violence. Further, there is no research exploring GPs’ role in supporting families through family court in the context of domestic violence.

**Methods:**

This study used qualitative methods. Fifteen GPs participated in individual in-depth interviews. The interviews were audio recorded with consent, transcribed verbatim and thematically analysed.

**Results:**

The majority of participants were female GPs working in metropolitan settings. Four themes were generated from the data: *on different planets, witnessing legal systems abuse, weaponizing mental health in family court* and *swinging allegiances.* Participants had negative perceptions of family court and felt that it operated on a different paradigm to that of general practice which caused difficulties when supporting patients. Participants supported survivors through instances where the court was used by perpetrators to further their abusive behaviour or where the court acted abusively against survivors. In particular, perpetrators and the family court used survivors’ mental health against them in court proceedings, which resulted in survivors being reluctant to receive treatment for their mental health. Participants struggled with their allegiances within their patient family and usually opted to support either the mother, the father, or the children.

**Conclusions:**

Implications of these findings for GP training are evident, including curriculum that discusses the intersection of mental health diagnoses and legal proceedings. There may also be a place for health justice partnerships within general practice.

## Background

Domestic violence or intimate partner violence is defined as any behaviour from an intimate partner that causes physical, sexual or psychological harm [1]. While domestic violence can affect both men and women, it is a gendered phenomenon with men using abusive behaviours towards their female partners frequently and severely, with one-third of women effected worldwide [1]. In an Australian context, one in six women and one in 16 men have experienced physical or sexual violence by a current or former partner [2]. Those who have experienced domestic violence can suffer a myriad of short and long-term physical, sexual and reproductive and psychological health issues [1].

Given this, survivors of domestic violence use healthcare services at an increased rate compared to the general population [1]. Health practitioners are often the first professionals to be trusted with a disclosure of abuse [2]. In general practice where there is an ongoing relationship with families, a general practitioner (GP) might support survivors and their children before, during or after they separate from their partners [3]. Many practitioners do not feel ready to address this complex issue of domestic violence [4], particularly if they are seeing all members of the family including the perpetrators [5].

Going through family court proceedings can have negative impacts on the health of victim/survivors [6] and on children and young people’s mental health [7]. Some of the negative health impacts stem from legal systems abuse, or the way in which the legal system contributes to extending an intimate partner’s abusive behaviour [8]. Thus, survivors report experiencing re-traumatisation and secondary victimisation, and long term psychological and physical health issues, including Post Traumatic Stress Disorder, insomnia, and depression [8–10]. Survivors often attend GPs during this time with these symptoms looking for support [11]. Despite this, there is no research exploring GPs’ role in supporting families through family court in the context of domestic violence. Such research is important for developing an evidence base on which training for health professionals supporting survivors can be built. The following study fills this gap as it aims to explore Australian GPs experiences of supporting families through domestic violence in the context of family court proceedings.

## Methods

### Study design

We conducted individual semi-structured interviews given the sensitive nature of the topic of domestic violence. Individual interviews can allow for a deep exploration into experiences, leading to a rich understanding of their experiences [12]. This study was underpinned by a constructivist paradigm which contends that there is no single truth, but truth in each person’s experience [13]. The study followed a phenomenological approach [13].

### Participants and recruitment

GPs were recruited via purposive, convenience and snowball sampling through existing networks at the Department of General Practice, University of Melbourne. GPs were eligible to participate if they had supported women who had experienced domestic violence and family court processes. GPs were recruited by direct email, particularly if they had a known interest in DV and a GP specific Facebook group on violence against women. GPs were invited to express interest and were emailed a plain language statement and a consent form before setting up an interview time that was convenient for participants.

### Data collection

The interview guide began by asking GPs to recount their experiences of supporting survivor women through family court. The questions then went on to explore how participants responded to survivors, how confident they felt in their response, what barriers and facilitators existed for them and how they believed the response to survivors going through family court could be improved. The average interview length was 38 minutes. All interviews were audio recorded over Zoom due to COVID-19 restrictions or because of participants’ personal preference. The second author conducted the first nine interviews, and the first author conducted the last six interviews. All interviews were transcribed verbatim by either the second author or a commercial transcription agency. All transcripts were de-identified during the transcription process. Pseudonyms were chosen for participants.

### Data analysis

The interview transcripts were imported into NVivo 12 (QSR International) for reflexive thematic analysis using an inductive and deductive approach (13). After a familiarisation process, the first author engaged in line-by-line coding to develop preliminary themes which were reviewed by the research team to create a coding framework. The research team included two survivors of DV who were able to use their lived experience to inform the analysis, which provided the deductive aspect of the analysis. The coding framework was applied to the remaining transcripts, with the survivors and the last author reviewing the application of the coding framework to transcripts to ensure rigour. This process resulted in the number and content of the themes shifting during the period of analysis as the themes were iteratively developed.

### Ethical considerations

Measures were taken to ensure the survivors on the research team felt safe to participate in data analysis. The survivors were given a summary of the project so they could make an informed choice about being involved in data analysis and a meeting was held to discuss safety, integrating trauma informed principles into the analysis process, power imbalances and promote inclusion. Ethics approval was obtained from the Human Ethics Advisory Group, Department of General Practice, The University of Melbourne (Ethics ID: 1955444.1).

## Results

Fifteen GPs participated and the majority of participants were women practicing in metropolitan areas (see Table 1). Reflexive thematic analysis generated four themes: *on different planets; witnessing Legal Systems abuse; weaponizing mental health in family court;* and *swinging allegiances.* Table 2 gives an overview of the subthemes relevant to each theme.

**Table 1:** Participant Demographics

Pseudonym	Gender	Age Group	Years Practicing as a GP	State	Practice Setting
Daisy	Female	60–69	35	Victoria	Metropolitan
Holly	Female	30–39	10	Victoria	Metropolitan
Violet	Female	30–39	9	Victoria	Metropolitan
Ash	Male	50–59	25	New South Wales	Rural/Regional
Hyacinth	Female	70–79	38	New South Wales	Metropolitan
Jasmine	Female	30–39	11	Tasmania	Metropolitan
Lilly	Female	50–59	20	Victoria	Metropolitan
Poppy	Female	60–69	40	New South Wales	Metropolitan
Marigold	Female	30–39	4	Victoria	Metropolitan
Rose	Female	60–69	45	Victoria	Metropolitan
Clementine	Female	40–49	20	Victoria	Metropolitan
Hazel	Female	50–59	30	Victoria	Metropolitan
Oliver	Male	40–49	19	Victoria	Metropolitan
Ivy	Female	50–59	26	Victoria	Metropolitan
Iris	Female	40–49	7	New South Wales	Rural/Regional

**Table 2:** Themes and subthemes

Theme	Subthemes
On different planets	• Mismatching paradigms between general practice and family court • Being unable to understand a system that feels foreign • The legal system being a roadblock for accessing care
Witnessing legal systems abuse	• Perpetrators using family court to further their abusive behaviour • Family court participating in abuse
Weaponizing mental health in family court	• Gaslighting by family court and perpetrators • Women feeling prevented from seeking help
Swinging allegiances	• GPs feeling loyal to their patient • Questioning the validity of equal parenting rights in the context of domestic violence • Persevering in their support role

### Theme 1: on different planets

All participants expressed feeling a profound difference in the paradigms that governed their practice versus those that dictated practices in family court. This difference left GPs in this study feeling as if they and the legal system were operating on different planets. This theme explores GPs’ views and knowledge of the family court system and how this informed and limited their response to survivors going through family court.

Participants’ perception of family court was resoundingly negative, even outside the context of domestic violence:

*‘I think the family court is universally…*[the] *most horrific, horrific, terrible, awful, devastating experience. It sucks out your soul. The actual court process… is so adversarial. It is brutal and people lie and set up each other and it’s so nasty… It’s just, it just rips people apart. It sets people against each other on probably the most emotional issue you can possibly imagine, which is your family…* (Ash)

Many GPs in this study recounted their patients’ stories which reinforced this negative perception. For example, Daisy related a story that left her ‘flabbergasted’ where her patient, Jo* (pseudonym given to the patient by Daisy), was taken to court by her ex-partner to gain unsupervised access to their children. Jo explained to Daisy that her ex-partner had previously physically abused her and that there was also a possibility of him sexually abusing their daughter. Jo’s court experience began with her being denied legal aid, as it was given to her ex-partner instead. Daisy then explained the outcome of Jo’s family court experience:

Anyway, the judge said… that Jo* had made it all up… She [the judge] *didn’t just change the ruling from the father could have unsupervised access, right there and then she took the children off the mother into the father’s full care…even as a medical professional… I was shocked actually… like don’t expect justice necessarily.* (Daisy)

Stories like these made participants feel helpless in giving advice to patients about going through family court. They struggled to find practically useful advice to give to patients as they felt they did not have the appropriate legal expertise. Thus, GPs in this study felt they could only provide emotional support:

*But, but the issue for me, as the GP, has been, I’m not legally trained. I can’t advise her on the legal stuff, clearly. …and I think that that makes it harder for me, I mean all I can do is offer her support and I can believe her, I can make her feel validated and give her a safe place that she can come to.* (Holly)

Even those participants with more training who practiced regularly in this area lacked confidence in their response because they were trying to understand a system that felt foreign to them:

*I do this every day and I still lack confidence because I’m often trying to project into the heads of how the legal system works, which I really don’t understand… there’s not a partnership between the solicitors and the doctors and this is a Medicare problem. I wish there was a way … where I could get together with the lawyer and me and the psychiatrist or the psychologist, so we could have a three-way conversation about what can and can’t be done.* (Hazel)

The issue of the legal system feeling foreign was increased when GPs found themselves supporting patients of culturally and linguistically diverse backgrounds, where the cultural divide made it difficult for patients to understand the Australian legal system’s decisions. Here, GPs fell into an educational role that complemented their emotional support role as they tried to explain to these patients how a Western legal system would differ from that of their own country:

*Another* [patient], *also a refugee background…he* [the patient] *kept saying ‘but I’m the father…I’ve got to see my children’ and it’s sort of a very cultural inability to understand how anyone apart from the parents can have a higher authority when it comes to the children…And lots of my patients I see, the challenge is, you are trying to gulf a bridge of understanding…* (Oliver)

Because of participants’ perception of the legal system and their lack of expertise, many found themselves working off their own experiences of family court, or those experiences they’d heard about, to provide practical advice to their patients. Some stated that they were reluctant to advise their patients to go through family court when they were having custody issues. Others supported their patients’ decision to go through family court with the caveat that ‘things will get worse before they get better’ (Clementine). The negative tone of this advice was powered by the belief that the ‘legal system is broken’ (Iris). This extended to the way in which the legal system interacted with the health system, where the legal system became a roadblock for accessing health services:

*Yeah, that comes up a lot, how to access the health system when there are court issues, because I think people* [within the health sector] *get really scared about the fact that this might have potential court issues. They* [health service providers] *keep going, oh it’s really sub-specialised, we can’t manage it. So, it actually throws out even further barriers.* (Clementine)

Clementine highlights that differences between the health sector and the legal sector are such that she perceived reluctance in providers in the health system to engage with survivors that have the added complexity of legal issues. This preserves the siloed nature of each of these sectors and creates further barriers to accessing support for survivors and their families.

### Theme 2: witnessing legal systems abuse

This theme explores the ways in which perpetrators used family court as a tool to further their abusive behaviour and how the court sometimes participated in the abuse. Most GPs in this study spoke of the way in which the legal system was used by perpetrators to perpetuate ongoing abuse similar to what survivors had previously escaped. The examples participants used to illustrate how perpetrators used the court to further their abuse patterns demonstrated several strategies aimed at presenting survivors to the court in a way that disadvantaged them. This was seen in Holly’s account of her patient having to be interviewed by her abuser in court:

*To have to be interviewed by her abuser, I mean, how can she possibly say what she’s truly thinking … when she’s got the person who had power over her, all the time, being the one hammering questions at her? And also, tearing her down and making her look bad in front of the court … she felt that the court probably got the wrong picture of what she’s truly like … he sort of got his say more than her and that perhaps she felt not completely believed…* (Holly)

In these instances, participants perceived that survivors felt that the truth of their experiences was undermined by perpetrators. Participants then perceived that survivors felt that the perpetrator’s portrayal of them in court damaged their bid for custody:

*But he uses that* [mild distress from major surgery] *in the court to say that she is incapable of having the children…it’s a control question. I don’t think it’s about the kids… I hear this story so often.* (Hazel)

In fact, most participants suggested that, from their point of view, perpetrators seemed to have little interest in their children prior to family court. However, this changed when the court became involved:

*I have seen perpetrators demand access to their children whereas prior to that they couldn’t give a toss about the kids…but as soon as it becomes a battle between who’s the good parent and who’s the bad parent, immediately they want access… That actually is additional violence…It’s an emotional and psychological violence that is perpetrated on the women.* (Rose)

Perpetrators’ need of control sometimes extended towards denying their children the psychological help they needed. In these cases, perpetrators seemed to use the legal power they had in their parental rights to prevent their children from accessing services:

*Another thing that they* [the children] *weren’t allowed to do during that process was to see their psychologist for support, so that was denied… everything had to be done with his* [the perpetrator’s] *approval. So, he was happy to see the court psychologist and just to tell the psychologist about his suffering. However, they* [the children] *couldn’t go off and do it themselves even though they were self-harming, they couldn’t even report that or that they were suicidal.* (Ivy)

Hazel claimed that this might be because perpetrators didn’t want their children speaking out against them in a way that could be used in court.

*…dad will not allow him to see a paediatrician, to see a psychologist to get treatment…and I think it is a power question.* [Imitates perpetrator] *‘We don’t want the children mentioning to a health professional what’s happening at home, so therefore we don’t want the kids seeing a health professional.’* (Hazel)

While the above are examples of the court being used for perpetrators’ gain, there were also several instances of the court actively participating in behaviours that are considered abusive. Violet recounted an experience from a patient who had experienced sexual violence post separation and a resultant pregnancy, which had to be terminated and the case ended up in court:

*…she was at the court and the interpreter they used, because she understands English very well, but her spoken English isn’t very good, and she said the interpreter was just interpreting the completely incorrect things… she’d say something and he’d say something not even related to what she said. And she kind of left the court not having done what she’d wanted to do…* (Violet)

In this way, the court was perpetuating the effects of abuse where the survivor can feel unheard or disempowered. Further contributing to this feeling is the experience of ‘judge lotto’, where a couple of participants stated that they or their survivor patients was allocated a judge who was ‘known to be difficult’ (Ivy) or ‘known for being particularly harsh’ (Iris) towards survivors in cases of domestic violence. Iris, a GP with her own experience of going through family court in the context of domestic violence, explains:

*I feel like there’s a real disconnect between what we say to women when they come in and disclose violence and what actually happens to them when they leave and how the system supports them. Because I was horrified by my own experience of the way you’re treated by the court….I felt really empowered by the women’s services…just before I left. But then I felt completely alone in the legal system. I felt like everyone was questioning the reality... like I’d made it all up to be a difficult person and to make my ex-husband’s life hard.* (Iris)

Thus, some participants viewed the court as having an active role in the perpetration of domestic violence. Ivy, after relating an experience in which she felt her patient’s domestic violence experience was silenced by lawyers who were dealing with a judge who had an equal custodial rights stance, explicitly stated:

*I think the court was used as a form of family violence really as a perpetrator.* (Ivy)

This was echoed by Hyacinth, who reflected on a common thread of the survivor journeys she had supported through the courts:

*It* [the legal system] *continues to perpetrate violence against the victims. It becomes part of the system that continues to perpetrate violence against the victims.* (Hyacinth)

Both these quotes demonstrate that these GPs see the court as active perpetrators of domestic violence.

### Theme 3: weaponizing mental health in family court

Many participants conveyed survivors’ concerns about seeking help for their mental health for fear that it may be used against them in family court. This theme continues to look at how family court perpetuates abuse but focuses specifically on how participants felt mental health was weaponised in family court.

Many GPs in this study witnessed their survivor patients being made to feel as if they had ‘made it all up’ (Iris, Daisy) throughout the court process. This element of gaslighting by the court and by perpetrators was common to many of the participants’ experience of supporting survivors through family court:

*Because he’s* [the perpetrator] *got that narcissistic personality, everyone sees him as a very powerful, competent, capable person who unfortunately is married to this mad wife. That’s a dynamic I see a lot, of re-casting women into neurotic, depressed, incapable, so causing the mental illness and then blaming the women for the mental illness which is a reason why they shouldn’t get custody. Not necessarily that the men want the custody, but they don’t want the women to have it. I would see that two out of three, I reckon.* (Hazel)

Because survivors knew that their mental health may be used against them in court, they felt they were prevented in seeking treatment for their mental health:

*What the family court has also done is, when people, when the victim has been to a GP, got a mental health plan and gone to a psychologist for help and support, that’s been used by the perpetrator to say that the victim is mentally unwell and not capable of looking after their children. So, this has been used also against victims in the family court…so the patient who is the victim is wanting not to do anything…which might jeopardise her access to her children* (Hyacinth)

*…women will not seek treatment, so they’ll say, I can’t be on antidepressants because he’ll use that against me in court. So, I often have to negotiate my treatment, so they look - inverted commas – sane in court…sometimes I’m saying…if they’re going to discriminate, they’re going to discriminate. It’s more important we keep you alive.* (Hazel)

Additionally, some patients requested that GPs left certain things off their patient file in case the perpetrator’s legal counsel were to subpoena their GP’s notes:

*…and if he* [the perpetrator] *subpoenaed the notes, if this partner tried to get this information from the file, that he might use that against her in court is what basically was what she said to me. That’s interesting because obviously even though the relationship has ended there’s still fear of control.* (Marigold)

Patients fearing that their mental health may be used against them in court made it difficult for GPs to provide care to their patients to the best of their ability. It prevented GPs from completing aspects of their job, like prescribing antidepressants or writing clinical notes, that may be essential in providing and accessing the best care for their patients. Beyond that, some GPs expressed a need to stop mental health services for children being blocked by lack of consent from perpetrators:

*If we were serious, we would be considering how to mandate mental health and physical health care for children who are victims of intimate partner violence, so it can’t be blocked by the other side. That’s horrendous…children should not have their health be a bargaining chip and it is, often.* (Hazel)

### Theme 4: swinging allegiances

All GPs were supporting their patients in the context of their whole family. This theme examines how GPs navigated seeing a family going through domestic violence and court proceedings by exploring the loyalty they felt to their patient, their views on the validity of equal parenting rights in the context of domestic violence and the confusion this created in choosing who to support, and how participants actualised this support.

Often, GPs seemed compelled to be loyal to one particular aspect of the family unit whether that be the mother, the father or the children. GPs would often choose to support the person they felt was their primary patient or the person who they felt was most victimised by the circumstances:

*I explained to him* [the perpetrator] *that it was nothing to do with him and me and our relationship as doctor-patient, it was completely to do with the fact that there are guidelines to say that it’s inappropriate in the circumstances for me to be looking after both people, and therefore he did need to find another doctor.* (Holly)

*I’ve also seen the opposite where the female partner has accused the male partner of violence when I have not been convinced of that…In this instance, I knew both the parents and… I found her behaviour to be very poor and he was the one who was suffering the verbal and the emotional abuse.* (Rose)

*I was involved because I was seeing the children. She was a patient of the practice but not my patient. I was the doctor for the children and I was really concerned for the children’s wellbeing.* (Clementine)

In other cases, some GPs like Jasmine already felt a connection and loyalty to one member of the family with whom they had interacted most and felt conflicted when the other parent asked for information about the health of their children:

*I think one of the things that does come up, like today, is that you’ve known the mother and the child and then all of a sudden, the father’s wanting to make contact and ask questions. And that’s awkward and your allegiance, your loyalty is sort of with the mother at that point but medically and legally you have to, you know, it’s the father, you can’t not engage. So, that’s often what we get…just that very awkward position of being in the middle of a parental dispute and knowing only one side of the story.* (Jasmine)

Several GPs contemplated the idea of equal parenting rights in the context of the law and domestic violence. Poppy wondered if the assumption of equal access being better for the child was the right fit in cases of domestic violence:

*There might also be underlying assumptions like every child deserves access to both their parents. It’s better for them….So the rights approach doesn’t always fit with the on the ground happenings.* (Poppy)

Meanwhile, Clementine considered whether any or all abusive behaviour warranted complete restriction of a parent’s right to see their child:

*She’d tell everybody how awful this father was… but does that justify no longer ever being able to see your children? I’m not sure that actually does… I just feel like it’s much more complex than black and white about this and I think it can be damaging either way whatever you do. I don’t think that’s quite as simple for us to say that.* (Clementine)

Some participants went on to describe instances were they perceived that fathers were disadvantaged in family court because of the gendered nature of domestic violence:

*The children get custody usually with the mother rather than with the father, so men do have to fight a lot harder to get custody of their children and that’s the nature of the beast and I suspect rightly so because although there are female perpetrators of violence and sexual abuse, they do tend to be in the minority.* (Rose)

Thus, these participants seemed to highlight the subjectiveness of custody battles and therefore seemed to advocate for a more case-specific and nuanced approach towards custody and visitation rights in the context of domestic violence. These quotes also acknowledge the confusion these participants felt when supporting families going through family court. These GPs see the court battle from both sides, therefore it can be difficult to judge certain situations. In this way, allegiance may differ depending on the individual case and the way the GP perceives the patient family. Despite this, regardless of who participants felt a particular allegiance to, participants always persevered in their role to support their patients through family court:

*…the father again was my patient and yeah, so the issue here was as expected, mental health repercussions on the man regarding not being able to see his children,… not appreciating what the risks were… usually, there’s a lot of anger and swearing directed at various ex-partners… the challenge being just trying to get the conversation to back to where it should be and trying to get the person a little bit more grounded to find the way forward.* (Oliver)

*I do know that my presence in their* [the children’s] *lives is important. I’m able to give them unconditional positive regard.* (Poppy)

*…she* [the woman] *has said to me, thank you so much, you know, your support really helped me so, you know, that’s, that’s where it becomes rewarding.* (Daisy)

## Discussion

This is the first time that GPs have been interviewed exploring their experiences with supporting patients going through the legal system. GPs’ experience of supporting families through family court in the context of domestic violence was characterised by four themes: *on different planets; witnessing legal systems abuse; weaponizing mental health in family court; and swinging allegiances.* These findings illustrate the complexity and challenges of navigating post-separation support by GPs for survivors of domestic violence.

*On different planets* explored GPs’ negative view of the legal system and how it was reinforced by the negative experiences their patients had. The disconnect between the court’s actions and what GPs felt should be done created a distance between the legal system and the health system, often highlighting that the two operated on different paradigms. The guiding ethical principle for health professionals of ‘first do no harm’ [15] appears at times, from the GP perspective, to be in complete opposition to the practices that play out within the court system. General practice values patient centred care and focuses on listening, believing, and validating survivor experience and equitably supporting families through violence [16]. The legal system, powered by the adversarial system paradigm, is focused on questioning families’ experiences, and survivors’ stories might be doubted [17]. GPs in this study, who prioritise health and improving quality of life, struggle with family court’s methods which negatively impact the health of their patients.

One of the major ways the family court impacted health was through legal systems abuse, as discussed in *witnessing legal systems abuse.* Douglas (2017) defined legal systems abuse as ‘domestic violence perpetrated through litigation’ (p.85), where the court is used as an opportunity to continue abuse tactics [8]. Many GPs in this study described instances of the court being used as a tool in the perpetration of abuse [6]. Perpetrators used their legal rights to present survivors to the court in a way that undermined the truth of survivors’ experiences [6]. The court also caused harm through secondary victimisation [8, 9] by not providing survivors their legal right to be fairly represented in court, leaving them feeling dismissed and ignored. This is supported by recent research surveying women survivors’ experience of services which found that women and their children were ‘let down and unprotected’ by family court (Hegarty, 2022; p.76). *Weaponizing mental health in family court* also provided examples of secondary victimisation as survivors were often gaslit by the family court as perpetrators and the court itself re-casted survivors as mentally ill [6, 18]. This led to survivors being prevented from seeking much needed treatment for their mental health issues resulting from family violence [19]. This was frustrating for GP participants because, not only were they seeing their patients be disempowered and disbelieved by the court, but they were also impeded in providing their patients with the best possible care for their mental health issues.

Further impeding their ability to provide care was the confusion over who the GP felt they could best support, which was explored in *swinging allegiances*. Participants felt as if they had to choose who to support between family members. Sometimes this choice would be based on who the primary patient was for that participant and sometimes it was based on who the GP viewed as the victim. This led to internal debate about equal parenting rights, the way these rights were upheld in family court, and confusion about who to support when participants could see both sides of the court battle. Underlying this were the assumptions participants perceived to be present in court such as ‘the best interests of the child’ aligning with ‘both parents have the right to access their children’. The Family Law Act 1975 stipulates that family court’s focus is on the rights of children and their best interests, rather than on parental rights, but it does presume that both parents share equally in the responsibility for their child [20]. However, most GPs in this study felt that the court was concerned with upholding parental rights. Some GPs felt that this assumption was harmful in the context of domestic violence [21]. Recent research echoes these findings, with a study finding that women felt that the processes, culture and assumptions within the family court, including that of equal parenting rights, worked against them in court (Hegarty, 2022).

There were several limitations to this study. First, most participants identified as female and practiced in metropolitan settings, therefore these findings may not resonate with the broader group of GPs. Additionally, because most participants were recruited through existing networks, many participants had a previous interest in family violence research which may have introduced self-selecting bias. Despite these limitations, this paper reports on findings that were robustly analysed and informed by survivors in their capacity as experts by experience. This has led to potentially richer analysis that reflects survivor experiences through family court processes. Further, to the authors’ knowledge, this is the first study that explores GPs’ experiences in supporting those experiencing domestic violence and family court processes.

## Conclusion

There are major challenges for GPs to support survivors of domestic violence during family court processes. These challenges include a lack of understanding of the legal paradigm, perception of legal systems abuse against survivors (including use of survivor’s mental health) and finally being unsure who their allegiance is with as they see all members of the family. Implications of this for GP training are evident, including curriculum that discusses the intersection of mental health diagnoses and legal proceedings, as well as opportunities to further knowledge on family court matters where appropriate. Health justice partnerships [22] are available in hospitals and there may be a place for similar close working arrangements between GPs and family court lawyers for individual patients. Judges need to be trained in family violence to understand the complexity of survivors’ experiences. For survivors, reform of the family court system is urgent [18].

## Data Availability

The authors declare that data supporting the findings of this study are available within the article.
